# No Clear Association between Impaired Short-Term or Working Memory Storage and Time Reproduction Capacity in Adult ADHD Patients

**DOI:** 10.1371/journal.pone.0133714

**Published:** 2015-07-29

**Authors:** Christian Mette, Marco Grabemann, Marco Zimmermann, Laura Strunz, Norbert Scherbaum, Jens Wiltfang, Bernhard Kis

**Affiliations:** 1 LVR Hospital Essen, Department of Psychiatry and Psychotherapy, Faculty of Medicine, University of Duisburg-Essen, Essen, Germany; 2 Department of Psychiatry and Psychotherapy, University Medical Center Goettingen, Goettingen, Germany; Alexander Fleming Biomedical Sciences Research Center, GREECE

## Abstract

**Objective:**

Altered time reproduction is exhibited by patients with adult attention deficit hyperactivity disorder (ADHD). It remains unclear whether memory capacity influences the ability of adults with ADHD to reproduce time intervals.

**Method:**

We conducted a behavioral study on 30 ADHD patients who were medicated with methylphenidate, 29 unmedicated adult ADHD patients and 32 healthy controls (HCs). We assessed time reproduction using six time intervals (1 s, 4 s, 6 s, 10 s, 24 s and 60 s) and assessed memory performance using the Wechsler memory scale.

**Results:**

The patients with ADHD exhibited lower memory performance scores than the HCs. No significant differences in the raw scores for any of the time intervals (*p* > .05), with the exception of the variability at the short time intervals (1 s, 4 s and 6 s) (*p* < .01), were found between the groups. The overall analyses failed to reveal any significant correlations between time reproduction at any of the time intervals examined in the time reproduction task and working memory performance (*p* > .05).

**Conclusion:**

We detected no findings indicating that working memory might influence time reproduction in adult patients with ADHD. Therefore, further studies concerning time reproduction and memory capacity among adult patients with ADHD must be performed to verify and replicate the present findings.

## Introduction

Attention deficit hyperactivity disorder (ADHD) is associated with altered time perception performance in children [1–9] and adults [10–15]. Before examining time perception more closely, differentiating between short-term memory (STM) and working memory (WM) is warranted. According to Baddeley (2012) *“the term working memory (WM) evolved from the earlier concept of short-term memory (STM) and the two are still on occasion used interchangeably*.*”* In his review, Baddeley suggested using the term STM to *“refer to the simple temporary storage of information*, *in contrast to working memory*, *which implies a combination of storage and manipulation”* (see page 4 in [16]). Baddeley´s model provides the theoretical foundation for memory function as examined in the present study [16]. The central executive constitutes the primary component of this model, which contains three slave systems: the visual-spatial sketchpad, the phonological-loop and the episodic buffer. The central executive is the most complex component in the model, as it assigns the three slave systems to one of the three processing stages: attentional focus, storage and decision making. The visual-spatial sketchpad is responsible for storing, retrieving, and manipulating visual-spatial information, whereas the phonological loop stores and manipulates verbal information. The episodic buffer is a multimodal storage system that can save both visual and verbal information; this system was added to the original model in 2000, as a component was needed to provide buffer storage. Nevertheless, the capacity of all slave systems is limited [16, 17]. A review concerning the contributions of lesion studies to the comprehension of the neuroanatomy of WM showed that the structures of the prefrontal cortex and the parietal cortex are connected with WM [18] but that STM is associated with the prefrontal cortex[19, 20]. Memory processes are involved in several psychological and neuropsychological processes, such as executive function [21], and might also be important when interpreting psychological studies concerning human time perception [22, 23]. The authors of a study of time perception in healthy participants postulated the information processing model (IPM) [24], which is a psychological model that explains the perception of time intervals. The IPM is a pacemaker-accumulator model that includes three important stages in the perception of time: the clock stage, the memory stage, and the decision stage. The onset of a stimulus turns a switch that permits pulses from the pacemaker to transfer to an accumulator (clock stage). The pulses in the accumulator are *stored* in temporal memory storage (memory stage). Memory retrieval begins at the onset of the time stimulus, and a sample representing one of the time intervals is transferred to the comparator. A decision regarding whether to respond is initiated by comparing the discrepancy between the time values from the accumulator and those from memory storage [25–28]. The memory process in the IPM Model appears to assign a central role to active processing in WM rather than to the individual´s capacity of memory storage. Therefore, the role of STM in time processing is unclear in the context of the IPM. However, it can be assumed that perception and decision making concerning the durations of time intervals and memory processes are closely interconnected. This assumption is supported by neuropsychological studies of animal and human time perception [29, 30] and a study demonstrating that the hippocampus and the prefrontal cortex are crucial for the perception of time intervals [23]. Moreover, studies have shown that thalamo-cortical-striatal circuits are activated during interval timing tasks. These circuits include the structures of the basal ganglia, the prefrontal cortex and the posterior parietal cortex [19, 31, 32]. With respect to the mental disorder ADHD and its related neuropsychological deficits, studies of adult ADHD patients have revealed a deficit in WM performance [33, 34]. Moreover, studies showed a deficit in the phonological loop and the visual-spatial sketchpad in adult patients with ADHD [35]. Furthermore, fMRI studies of adult patients with ADHD showed decreased activity in the temporo-occipital and prefrontal cortices [36, 37], which are responsible for time and memory processing [19, 20, 31, 32]. These results support the hypothesized connection between time processing and memory. However, whereas several studies examined the deficits of adult ADHD patients in WM, studies examining STM in adult ADHD are scarce. To our knowledge, only one study has shown a deficit in the STM capacity of adolescents and adults with ADHD [38]. That study revealed that adolescents with ADHD exhibit reduced STM capacity and an increased discrepancy between STM capacity and verbal IQ [38].

Additionally, studies of adult patients with ADHD that have examined STM, WM and their possible link to the ability to reproduce time intervals are scarce. A study conducted by Barkley et al. (2001) examined a sample of 104 adult patients with ADHD and 46 control subjects using a retrospective time estimation task and a time reproduction task consisting of six time intervals (2 s, 4 s, 12 s, 15 s, 45 s and 60 s). The adult patients with ADHD did not exhibit longer time estimates on the time estimation task. Nevertheless, the ADHD patients committed more reproduction errors at the 15 s, 45 s and 60 s intervals and produced shorter reproductions at the 15 s and 60 s intervals compared with the healthy controls (HCs) [10]. The authors controlled for IQ differences and comorbid disorders but did not specifically examine measures of memory performance. A translational study investigated time discrimination, time duration estimation and time reproduction abilities of children with ADHD, children with dyslexia and adolescents with ADHD using 400 ms, 2 s and 6 s time intervals [39]. The children and adolescents with ADHD performed differently from the HCs on the discrimination task. A difference between the adolescents with ADHD and the HCs in time reproduction was detected for the 400 ms time interval. Further, the authors observed a higher variability among the adolescents with ADHD at the 2 s and 6 s intervals on the time reproduction task. No differences were observed in the mean performance at the 2 s and 6 s intervals among the adolescents with ADHD. The authors also reported that STM and WM performance predicted performance on the duration discrimination task but not on the duration estimation task [39]. However, the findings of Toplak and colleagues were limited to adolescents, and an adult sample is needed to generalize these findings regarding the possible association of time reproduction capacity with STM and WM in adults with ADHD. The scope of our study was to investigate time reproduction in the range of seconds in adults with ADHD using six time intervals selected from the current literature and to determine the possible association of time reproduction performance with STM and WM.

The following hypotheses were examined in this study. According to the recent literature, patients with ADHD would perform worse on time reproduction tasks, as well as STM and WM tests, than HCs (H1). In the case of an association between memory performance and the ability to reproduce time intervals, we postulated an association of time reproduction with STM and WM. Specifically, the ADHD patients who performed worse on the STM/WM tests would also perform worse on the time reproduction task compared to the HCs (H2).

## Materials and Methods

### Study design

The study was conducted using an experimental group-control group design composed of three groups. The first group consisted of adult patients with ADHD who were regularly receiving methylphenidate (MPH) medication (MPH+); the second group consisted of patients with ADHD who were not receiving MPH or any other medication (MPH-); and the third group was a HC group. The necessary sample size (n) as a function of the power (1-β), the significance level (α) and the effect size (f) was calculated using the statistical power analysis program G-Power 3 [40]. The expected a priori power was set at 1-β = .80, and the significance level was set at α = .05 for three groups. Based on previous results concerning time reproduction among adult ADHD patients in the literature [10, 15, 39], we expected a medium effect size (f = .35). A required total sample size of n = 84 was calculated.

### Sample

We recruited 59 patients with confirmed diagnoses of ADHD from a pool of adult patients with ADHD who were receiving treatment at the ADHD outpatient clinic of the Department of Psychiatry and Psychotherapy, University of Duisburg-Essen, and 32 healthy adult controls (M_Age_ = 31,28; SD_Age_ = 7,14) were recruited via postings in various university and non-university settings. The exclusion criteria for the patients with ADHD included other psychiatric disorders, such as co-morbid depression and mood disorders or substance abuse, and the current use of psychotropic medications other than MPH. Patients with ADHD were diagnosed by trained psychiatrists and psychologists at the ADHD outpatient clinic according to the DSM-IV-TR criteria. To control for covariates, questionnaires regarding current difficulties related to attention, hyperactivity and impulsivity (ADHD self-rating questionnaire, ADHS-SB; Wender Utah Rating Scale, WURS-K) [41, 42], state and trait anxiety (State-Trait Anxiety Inventory, STAI X1 and X2 [43]), depressive symptoms (Beck’s Depression Inventory, 2^nd^ edition, BDI [44]) and the baseline levels of impulsivity (Barrett Impulsiveness Scale, BIS [45]) were administered to all subjects. Participants with diabetes; obesity; schizophrenia; current substance abuse; or a history of drug abuse, mood or anxiety disorder or any other chronic medical condition were excluded.

Participants with an IQ below 85 were not included in the study. IQs were estimated using the “picture completion” and “similarities” subtests of the Wechsler Intelligence Scale for adults [46] by combining the scores on these two subtests (WIP (S/PC)). Furthermore, we administered the B version of the German Mehrfachwahl-Wortschatz-Intelligenz-Test (MWT-B) [47], which is a multiple-choice word test that resembles the English spot-the-word test. Both IQ estimates are displayed in Table 1.

**Table 1:** Demographic characteristics of the ADHD and healthy control groups.

Variable	ADHD MPH+	ADHD MPH-	Healthy controls	H-test
	M (SD)	M (SD)	M (SD)	*p-value*
	*(n = 30)*	*(n = 29)*	*(n = 32)*	
Age (years)	M = 34.73 (9.08)	M = 34.72 (10.40)	M = 31.28 (7.14)	*n*.*s*.
Years of education	M = 16.77 (3.50)	M = 15.90 (2.50)	M = 16.84 (1.70)	*n*.*s*.
MWT-IQ	M = 109.30 (10.60)	M = 107.80 (17.0)	M = 113.20 (18.80)	*n*.*s*.
WIP(S/PC)	M = 109.30 (7.95)	M = 109.60 (7.00)	M = 112.60 (8.20)	*n*.*s*.
WURS-K	M = 38.03 (13.40)	M = 38.28 (13.44)	M = 9.88 (8.21)	*p <* .*001*
ADHS-SB	M = 30.60 (9.84)	M = 38.03 (13.40)	M = 7.16 (5.43)	*p <* .*001*
STAI X1	M = 44.97 (8.92)	M = 44.31 (9.60)	M = 30.88 (5.93)	*p <* .*001*
STAI X2	M = 49.27 (9.55)	M = 48.59 (9.59)	M = 32.88 (5.58)	*p <* .*001*
BIS	M = 76.80 (11.58)	M = 75.57 (12.72)	M = 53.88 (7.98)	*p <* .*001*
BDI-II*	M = 12.87 (9.10)	M = 12.03 (8.53)	M = 4.22 (4.46)	*p* = .*01*

The characteristics of the three analyzed groups are shown. Data regarding age, years of education, Wechsler Intelligence Scale score, mean IQ (based on the similarity subtest and the picture completion subtest of the WIP (WIP(S/PC))), multiple-choice word test (MWT IQ) score [this test resembles the English spot-the-word test], the Wender Utah Rating Scale (WURS-K) score, the ADHS self-rating questionnaire (ADHS-SB) score, the State Trait Anxiety Inventory (STAI X1 and STAI X2) score, the Barrett impulsiveness scale (BIS) score and the Beck’s Depression Inventory (BDI-II) score are provided. ADHD MPH+: patients with ADHD who were taking methylphenidate; ADHD MPH-: patients with ADHD who were not taking methylphenidate; M: mean; SD: standard deviation; n: sample size; *considered as a covariate in the ANOVA.

The patients in the MPH+ group had been treated with long-acting MPH formulation for at least one year prior to the start of the present study. The patients with confirmed diagnoses of ADHD were divided into two subgroups according to the status of MPH treatment. The patients in the MPH+ group (n = 30; M_Age_ = 34.73; SD_Age_ = 9.08) had been treated with a long-acting MPH formulation (M = 35.7 mg; SD = 16.1 mg) for at least one year, and the patients in the MPH- group (n = 29; M_Age_ = 34.72; SD_Age_ = 10.40) had not received any prior pharmacological treatment. These groups were comparable in terms of group size and gender (χ^2^ = .21 p > .05) and did not differ significantly with respect to age, formal education or estimated IQ (Table 1). This study was evaluated and approved by the Ethics Committee of the Faculty of Medicine of the University Essen-Duisburg and was conducted in accordance with the Declaration of Helsinki.

### Time reproduction task

The time reproduction task was compiled using the PYTHON programming language, version 2.7.3. (open source license; Python Software Foundation 2012; www.python.org) and was executed on a laptop PC (Lenovo Think Pad, Intel core i5) running Lubuntu Linux equipped with system kernel version 3.2.0-24-generic (open source license, Ubuntu Foundation 2012, www.ubuntu.com). Time reproduction in the range of seconds was tested using a computerized time reproduction task consisting of six time intervals (1 s, 4 s, 6 s, 10 s, 24 s and 60 s) that have frequently been used in previous research [5, 10, 14, 15]. The time reproduction task was administered visually, and standardized verbal and visual instructions were used. Thereafter, each of the six time intervals was randomly presented 10 times on a computer screen. After the presentation of an exclamation mark (100 ms), a non-moving classic yellow smiley face was projected onto the computer screen for a specific time interval (e.g., 10 s) and was then removed. The subjects were instructed to reproduce the time interval by pressing the left (ctrl) key within 1000 ms after the smiley face disappeared and to continue pressing the left (ctrl) key for the same duration that the classic smiley face had appeared on the screen. The participants were instructed to release the left (ctrl) key when they perceived that they had matched the presented time interval. After an inter-trial interval of 100 ms, a new trial began. If the participants did not press the left (ctrl) key within 1000 ms, the next trial began after the inter-trial interval.

The computerized task was administered after the completion of the test battery, which included the questionnaires mentioned above and the assessment of memory performance. The order of task administration was identical for all participants. Furthermore, to control for confounding variables, the testing room did not contain any external clocks. The subjects were asked to turn off their mobile phones and to store their watches in their pocket or handbag. Further, the participants were explicitly instructed not to count during the time reproduction task. In addition, after completing the time reproduction task, all participants were asked whether they complied with the “no-counting” instruction; all participants confirmed compliance. For statistical analyses, the raw time reproduction ability in terms of the medians, means, standard deviations and reaction times for the reproduced durations at all six time intervals were calculated. Furthermore, we calculated the variability in time reproduction using the coefficient of variation (cv = SD_time_/M_time_) of the reproduced durations for all six time intervals.

### Assessment of memory performance

To assess the STM and WM performance of the subjects, the German version of the digit span forward, digit span backward, block span forward, block span backward and letter-numbering sequence subtests of the Wechsler Memory Scale [48] were administered.

### Data analyses

All statistical calculations were performed using SPSS version 22.0 (IBM). To analyze the comparability of the MPH+, MPH- and HC groups, a χ^2^ test was applied. To test the data for normal distribution, Kolmogorov-Smirnov tests were performed. The Kolmogorov-Smirnov tests revealed non-normal distributions for all dependent variables (*p* = .001); the standard deviations (SDs) and the coefficients of variation for all six time intervals in the time reproduction failed to meet the criteria for parametric testing. Therefore, we performed non-parametric Kruskal-Wallis *H* tests. Furthermore, the between-group comparisons were performed using *H* tests. The significance level was set at *p* < .05. The SDs and the coefficients of variation (variability) for all time intervals (1 s, 4 s, 6 s, 10 s, 24 s and 60 s) were examined using H tests. *Post hoc* analyses were performed using the Mann-Whitney *U* test to determine the directions of the significant *H* test results.

All other variables exhibited normal distributions (*p* > .08). Therefore, we conducted parametric analyses of variance (ANOVAs) for the dependent variables from the time reproduction experiment with the exceptions of the SDs of the six time intervals. The following within-subject variables were considered in the ANOVAs: the medians of the reaction time and of the reproduced duration for each of the time intervals (1 s, 4 s, 6 s, 10 s, 24 s and 60 s). Additionally, group (MPH+ vs. MPH- vs. HC) was included as a between-subject factor in the ANOVAs. All ANOVAs were corrected using *post hoc* Bonferroni corrections and Greenhouse-Geisser adjustments. Bivariate correlations between memory performances and time reproductions were calculated according to Spearman's ρ. The α-level was set to *p* = .05.

The *H* tests revealed no significant differences between subjects with and without ADHD in terms of estimated IQ, the duration of education or age (all *p* > .05). Thus, the models were not adjusted according to intelligence, education or age. The results of the WURS-K, ADHS-SB, STAI, BIS and BDI-II questionnaires revealed significant differences between the ADHD patients and the HCs (Table 1). The statistical analyses revealed significant differences across groups on the WURS-K, ADHS-SB, STAI, BIS, and BDI-II questionnaires. Significant differences between the patients with ADHD and the HCs can be assumed in ADHD-related questionnaires such as WURS-K, ADHS-SB, STAI, and BIS. These differences were revealed based on statistical analysis. However, in contrast to general expectations, the BDI-II score also displayed a significant difference between the groups. Therefore, the BDI-II score was included as covariate in the analyses. Although the BDI does not diagnose clinical depression, we included the BDI score in the analyses to control for possible affective differences in the sample.

## Results

### Time reproduction

#### Raw scores

ANOVA of the raw scores of the reaction time and the reproduction duration for all time intervals (1 s, 4 s, 6 s, 10 s, 24 s, 60 s) revealed no significant differences between any of the groups (*p* = .05).

#### Variability

**Fig 1:**
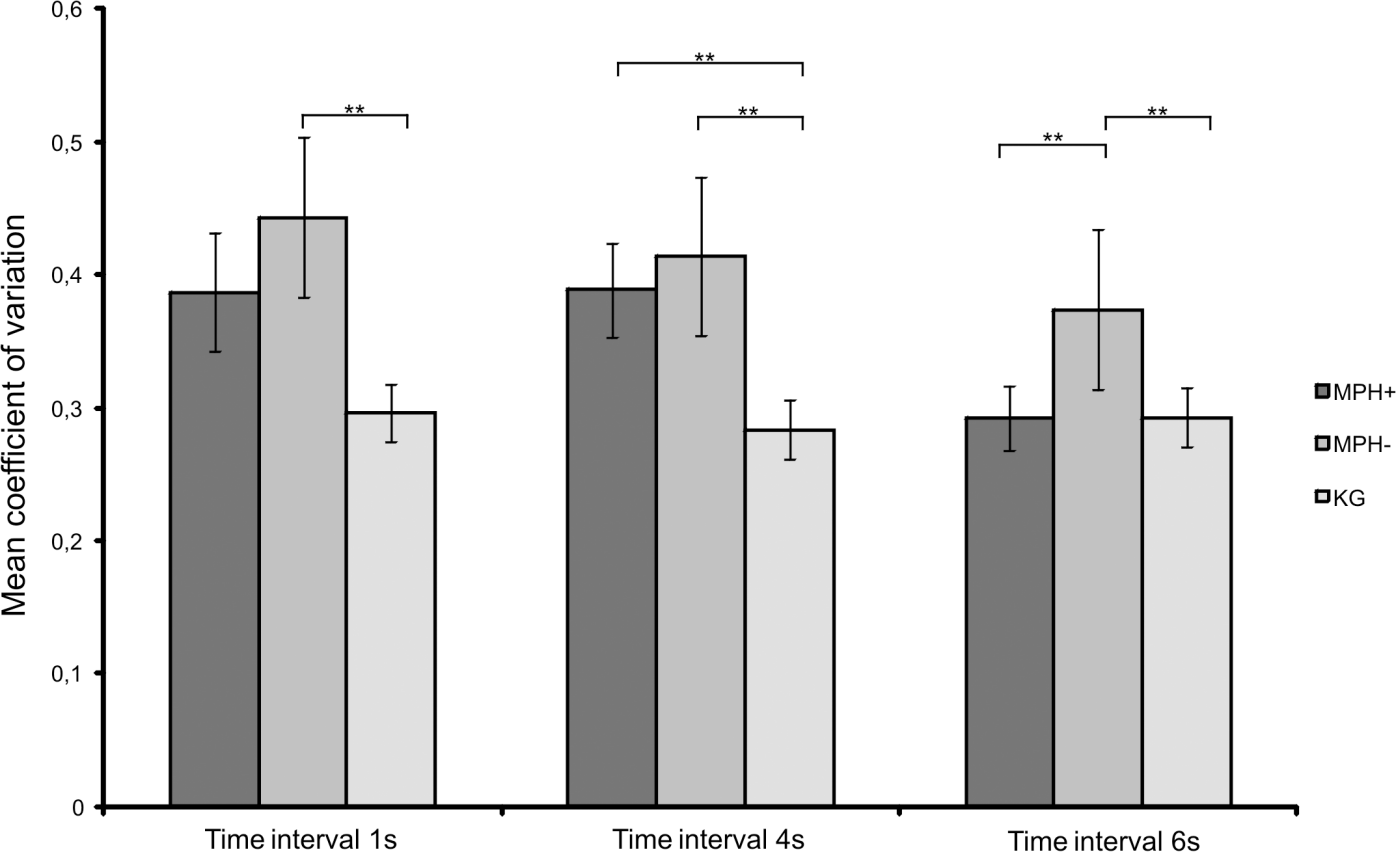
Time reproduction performance. Between-group differences in the coefficient of variation, indicating the variability in performance, for the reproduction times at the 1 s, 4 s and 6 s intervals for all groups. The means and standard deviations of the coefficients of variation for the 1 s, 4 s and 6 s time intervals are shown. MPH+: patients with ADHS who were taking methylphenidate medication; MPH-: patients with ADHD who were not taking methylphenidate; HC: healthy controls; *p < .05; **p < .01.

The *H* tests of the time interval SDs revealed significant differences in the SDs of the reproduction durations at the 1 s (*H*(2) = 6.14, *p* < .04), 4 s (*H*(2) = 8.70, *p* < .01) and 6 s intervals (*H*(2) = 8.54, *p* < .01) on the time reproduction task. The H tests of the coefficients of variation revealed significant differences for the 1 s (*H*(2) = 6.43, p = .04), 4 s (*H*(2) = 7.98, p < .01) and 6 s intervals (*H*(2) = 6.88, p < .03; Fig 1). No significant differences between the groups were found at any of the other time intervals.

### Short-term memory and working memory

The memory performance results are shown in Table 2.

**Table 2:** Assessment of the short-term and working memory performance of the ADHD and healthy control groups.

Variable	ADHD (MPH+)	ADHD (MPH-)	Healthy controls	*H test*	*Post hoc U test*
	M (SD)	M (SD)	M (SD)	*p-value*	
Digit span forward	M = 17.73	M = 17.10	M = 17.66	*n*.*s*.	*n*.*s*.
	(1.48)	(1.81)	(1.23)		
Digit span backward	M = 6.33	M = 5.76	M = 7.13	*p <* .*03*	*HC > MPH-*
	(1.76)	(1.78)	(1.64)		*U = 296*.*5 p <* .*001*
Block span forward	M = 8.33	M = 8.38	M = 8.38	*p <* .*002*	*MPH+ < HC*
	(1.42)	(1.56)	(1.56)		*U = 252*.*5 p* = .*009*
					*MPH- < HC*
					*U = 277*.*0 p* = .*006*
Block span backward	M = 8.23	M = 7.97	M = 9.44	*p <* .*002*	*MPH+ < HC*
	(1.75)	(1.50)	(1.50)		*U = 103*.*0 p <* .*001*
					*MPH- < HC*
					*U = 229*.*0 p <* .*001*
Letter-numbering sequence	M = 10.90	M = 9.45	M = 11.31	*p <* .*03*	*MPH+ > MPH-*
	(1.97)	(3.30)	(2.45)		*U = 293*.*5*, *p* = .*03*
					*MPH- < HC*
					*U = 298*.*5*, *p* = .*01*

The results of the memory assessments for the three analyzed groups are shown. Data are provided for the digit span forward, digit span backward, block span forward, block span backward and letter-numbering sequence tests. MPH+: patients with ADHD who were taking methylphenidate; MPH-: patients with ADHD who were not taking methylphenidate; M: mean; SD: standard deviation; n: sample size.

### Correlations

The overall analyses failed to reveal any significant correlations between any of the time intervals on the time reproduction task and WM performance (*p* > .05).

## Discussion

As expected, adult patients with ADHD exhibited worse memory performance than HCs. These results indicate that the patients with ADHD exhibited impaired STM and WM performance compared with HCs. With respect to neuropsychological deficits in attention, inhibition, memory and executive functioning, which have frequently been reported in patients with ADHD, the present results are consistent with the reported changes in STM and WM in this group of patients[49–52] and are in line with findings concerning the pathophysiology of adult ADHD [33, 53–61]. One study investigated WM deficits in adults with ADHD and showed impaired WM performance in these individuals. The authors administered the Letter-Numbering-Sequencing test and evaluated the digit span of the Wechsler memory scale. Patients with ADHD showed a lower score on the Letter-Numbering-Sequence test and the digit span subscale [62]. A recent fMRI study demonstrated a deficit in phonological WM and in associated brain areas. The authors administered an n-back task to 29 participants with ADHD and HCs. They detected a correlation between low WM performance and altered activation in the pars opercularis of the frontal inferior gyrus, which is associated with WM performance [63].

No general group differences in the raw time reproduction scores were found between the adults with ADHD and the HCs with the exception of the variability at the 1 s, 4 s and 6 s time intervals in the MPH- patients and at the 4 s and 6 s time intervals in the MPH+ patients. These results for the variability in time reproduction are in accordance with those of another study that demonstrated greater variability in time reproduction among children with ADHD [64]. Moreover, Mullins and colleagues only found enhanced variability at longer time intervals (from 36 s to 60 s), but not at shorter time intervals, among adults with ADHD. Furthermore, these results might be related to reports in the literature of absolute discrepancies in time reproduction among adults with ADHD. Valko et al. (2010) demonstrated greater absolute discrepancies between the mean reproduction times and the target intervals (for 4 s, 6 s and 8 s) in 33 children with ADHD; similar findings were observed for the 2 s, 4 s and 8 s intervals in 22 adults with ADHD compared with HCs [15]. In addition, similar effects were detected in young adults with ADHD. The authors investigated the raw scores and the discrepancies for the reproduction of time intervals in 104 young adults with ADHD and 64 HCs. These groups were found to differ in terms of the absolute discrepancy, indicating that the ADHD group exhibited higher variability in the reproduction of time intervals [10]. However, the results of the variability index are limited because there was no difference in the raw scores of the time reproduction experiment. Further, the difference in the variability scores across participants might be due to the adoption of distinct strategies by different individuals to increase their ability in the time reproduction task. Therefore, the results could confounded by such strategies, representing a limitation of the present study. Thus, further studies concerning time reproduction and the variability in time reproduction among patients with adult ADHD must be performed to verify and replicate the present findings.

Further, no general association between memory performance and the time reproduction results were observed. The relevant literature indicates that WM processes are impaired in adults with ADHD [35, 50, 56, 57]. Although we observed that WM was impaired in our patients with adult ADHD, no difference in the raw time reproduction test scores was found between the three groups. Therefore, we are unable to postulate a causal relationship between memory and time reproduction, and further studies are warranted to examine this possible relationship.

Aside from the findings regarding memory and the variability in time reproduction among adult patients with ADHD, our results are not in line with earlier or recent results of time reproduction performance in patients with adult ADHD [10, 11]. In their review, Noreika and colleagues reported 27 studies that have consistently shown a deficit in the reproduction of time intervals and three studies that reported inconsistent findings in patients with adult ADHD [11]. Thus, certain differential factors might have confounded the results of the present study. Differences in the BDI scores between the three groups might have influenced the results of the time reproduction task. However, the BDI was included as a covariate in the ANOVA to control for this possible influence. The present study offered an advantage in that it included an HC group that did not differ from the ADHD patient groups in terms of education, IQ or age, and our study design controlled for the effects of MPH. Further, it can be assumed that the investigated sample was somewhat representative, allowing the aforementioned conclusions to be drawn.

However, certain methodological limitations should be considered when interpreting these data. The WM tests employed do not provide high power compared to the 2-back or the 3-back task. Thus, the present results need to be verified or falsified in subsequent studies using n-back tasks to assess WM performance in adults with ADHD.

A further limitation might be that the subjects may have counted during the time reproduction task, thereby volitionally improving their time interval reproduction performance. Although all subjects reported that they did not count during the time reproduction task, the instructions not to count must be considered critically when interpreting the data. However, a methodological study demonstrated that the most effective way to prevent counting during time perception tasks is the “instruction method” [65]. The authors of that study compared three counting prevention methods: instructions not to count, the articulatory suppression method, and the administration of an interference task. Rattat and colleagues demonstrated that the instruction method most effectively prevented counting during time reproduction tasks [12, 60, 65]. Nevertheless, the possibility that the participants were not truthful with respect to counting during the time reproduction test cannot be ruled out. One group might have been more truthful than another group, which would have severely confounded and limited the findings of the present study. Therefore, investigations into the effects of counting and other strategies on the time reproduction performance of adults with ADHD are warranted in future studies.

We cannot postulate an association between memory and time reproduction due to the limitations mentioned above. Therefore, future studies that consider the abovementioned limitations are needed to draw general conclusions regarding the association between time reproduction and WM capacity. Although no significant difference in the ability to reproduce time intervals or association between time interval production ability and WM capacity was observed, the present study provides useful information for future research. The following questions should be investigated in future studies.

First, the specific effects of counting during time reproduction tasks in adult ADHD must be investigated. To our knowledge, no study has investigated the specific effect of counting in a sample of adult ADHD patients. Thus, it is warranted to investigate the heuristics of patients with ADHD compared to healthy controls. Because counting might represent only one of many possible strategies to improve time reproduction ability, further possible strategies such as buzzing or “mental singing” might also be considered as possible effective strategies. Therefore, such strategies must also be investigated before inferring effective strategies to prevent counting in time reproduction. Second, the lack of a confounding effect of these strategies on the WM capacity of the subjects must be demonstrated. Finally, studies need to be conducted to investigate the differences between STM and WM capacity according to time reproduction ability in adult ADHD patients by controlling for the limitations of the present study. Nevertheless, the preliminary results of the present study are encouraging for further research in the fields of time reproduction and WM capacity.

## References

[1] Gooch D, Snowling M, Hulme C (2011) Time perception, phonological skills and executive function in children with dyslexia and/or ADHD symptoms. J Child Psychol Psychiatry 52: 195–203. 10.1111/j.1469-7610.2010.02312.x 20860755 PMC3412207

[2] Hwang SL, Gau SS, Hsu WY, Wu YY (2010) Deficits in interval timing measured by the dual-task paradigm among children and adolescents with attention-deficit/hyperactivity disorder. J Child Psychol Psychiatry 51: 223–232. 10.1111/j.1469-7610.2009.02163.x 19754502

[3] Kerns KA, McInerney RJ, Wilde NJ (2001) Time reproduction, working memory, and behavioral inhibition in children with ADHD. Child Neuropsychol 7: 21–31. 11815878 10.1076/chin.7.1.21.3149

[4] McGee R, Brodeur D, Symons D, Andrade B, Fahie C (2004) Time perception: does it distinguish ADHD and RD children in a clinical sample? J Abnorm Child Psychol 32: 481–490. 15500028 10.1023/b:jacp.0000037778.61929.1b

[5] Meaux JB, Chelonis JJ (2003) Time perception differences in children with and without ADHD. J Pediatr Health Care 17: 64–71. 12665728 10.1067/mph.2003.26

[6] Plummer C, Humphrey N (2009) Time perception in children with ADHD: the effects of task modality and duration. Child Neuropsychol 15: 147–162. 10.1080/09297040802403690 18825522

[7] Rommelse NN, Oosterlaan J, Buitelaar J, Faraone SV, Sergeant JA (2007) Time reproduction in children with ADHD and their nonaffected siblings. J Am Acad Child Adolesc Psychiatry 46: 582–590. 17450049 10.1097/CHI.0b013e3180335af7

[8] West J, Douglas G, Houghton S, Lawrence V, Whiting K, Glasgow K (2000) Time perception in boys with attention-deficit/hyperactivity disorder according to time duration, distraction and mode of presentation. Child Neuropsychol 6: 241–250. 11992188 10.1076/chin.6.4.241.3140

[9] Yang B, Chan RC, Zou X, Jing J, Mai J, Li J (2007) Time perception deficit in children with ADHD. Brain Res 1170: 90–96. 17669375 10.1016/j.brainres.2007.07.021

[10] Barkley RA, Murphy KR, Bush T (2001) Time perception and reproduction in young adults with attention deficit hyperactivity disorder. Neuropsychology 15: 351–360. 11499990 10.1037//0894-4105.15.3.351

[11] Noreika V, Falter CM, Rubia K (2013) Timing deficits in attention-deficit/hyperactivity disorder (ADHD): evidence from neurocognitive and neuroimaging studies. Neuropsychologia 51: 235–266. 10.1016/j.neuropsychologia.2012.09.036 23022430

[12] Pollak Y, Kroyzer N, Yakir A, Friedler M (2009) Testing possible mechanisms of deficient supra-second time estimation in adults with attention-deficit/hyperactivity disorder. Neuropsychology 23: 679–686. 10.1037/a0016281 19702421

[13] Prevatt F, Proctor B, Baker L, Garrett L, Yelland S (2011) Time estimation abilities of college students with ADHD. J Atten Disord 15: 531–538. 10.1177/1087054710370673 20679155

[14] Seri Y, Kofman O, Shay L (2002) Time estimation could be impaired in male, but not female adults with attention deficits. Brain Cogn 48: 553–558. 12030506

[15] Valko L, Schneider G, Doehnert M, Muller U, Brandeis D, Steinhausen HC, et al (2010) Time processing in children and adults with ADHD. J Neural Transm 117: 1213–1228. 10.1007/s00702-010-0473-9 20821338

[16] Baddeley A (2012) Working memory: theories, models, and controversies. Annu Rev Psychol 63: 1–29. 10.1146/annurev-psych-120710-100422 21961947

[17] Baddeley A (2000) The episodic buffer: a new component of working memory? Trends Cogn Sci 4: 417–423. 11058819 10.1016/s1364-6613(00)01538-2

[18] Muller NG, Knight RT (2006) The functional neuroanatomy of working memory: contributions of human brain lesion studies. Neuroscience 139: 51–58. 16352402 10.1016/j.neuroscience.2005.09.018

[19] Zola-Morgan S, Squire LR (1993) Neuroanatomy of memory. Annu Rev Neurosci 16: 547–563. 8460903 10.1146/annurev.ne.16.030193.002555

[20] Cowan N (2008) What are the differences between long-term, short-term, and working memory? Prog Brain Res 169: 323–338. 10.1016/S0079-6123(07)00020-9 18394484 PMC2657600

[21] Lara AH, Wallis JD (2014) Executive control processes underlying multi-item working memory. Nat Neurosci 17: 876–883. 10.1038/nn.3702 24747574 PMC4039364

[22] Buhusi CV, Meck WH (2005) What makes us tick? Functional and neural mechanisms of interval timing. Nat Rev Neurosci 6: 755–765. 16163383 10.1038/nrn1764

[23] Meck WH, Church RM, Matell MS (2013) Hippocampus, time, and memory-A retrospective analysis. Behav Neurosci 127: 642–654. 10.1037/a0034201 24128354 PMC4629468

[24] Church RM (2002) A tribute to John Gibbon. Behav Processes 57: 261–274. 11948002 10.1016/s0376-6357(02)00018-9

[25] Church RM, Meck WH, Gibbon J (1994) Application of scalar timing theory to individual trials. J Exp Psychol Anim Behav Process 20: 135–155. 8189184 10.1037//0097-7403.20.2.135

[26] Gibbon AJ (1991) Origins of scalar timing. Learn Motiv 22: 3–38.

[27] Gibbon J, Church RM (1990) Representation of time. Cognition 37: 23–54. 2269007 10.1016/0010-0277(90)90017-e

[28] Gibbon J, Church RM, Meck WH (1984) Scalar timing in memory. Ann N Y Acad Sci 423: 52–77. 6588812 10.1111/j.1749-6632.1984.tb23417.x

[29] Meck WH (2005) Neuropsychology of timing and time perception. Brain Cogn 58: 1–8. 15878722 10.1016/j.bandc.2004.09.004

[30] Meck WH, Malapani C (2004) Neuroimaging of interval timing. Brain Res Cogn Brain Res 21: 133–137. 15464347 10.1016/j.cogbrainres.2004.07.010

[31] Lewis PA, Miall RC (2003) Distinct systems for automatic and cognitively controlled time measurement: evidence from neuroimaging. Curr Opin Neurobiol 13: 250–255. 12744981 10.1016/s0959-4388(03)00036-9

[32] Macar F, Lejeune H, Bonnet M, Ferrara A, Pouthas V, Vidal F, et al (2002) Activation of the supplementary motor area and of attentional networks during temporal processing. Exp Brain Res 142: 475–485. 11845243 10.1007/s00221-001-0953-0

[33] Kim S, Liu Z, Glizer D, Tannock R, Woltering S (2014) Adult ADHD and working memory: neural evidence of impaired encoding. Clin Neurophysiol 125: 1596–1603. 10.1016/j.clinph.2013.12.094 24411642

[34] Engelhardt PE, Nigg JT, Carr LA, Ferreira F (2008) Cognitive inhibition and working memory in attention-deficit/hyperactivity disorder. J Abnorm Psychol 117: 591–605. 10.1037/a0012593 18729611 PMC5221607

[35] Alderson RM, Hudec KL, Patros CH, Kasper LJ (2013) Working memory deficits in adults with attention-deficit/hyperactivity disorder (ADHD): an examination of central executive and storage/rehearsal processes. J Abnorm Psychol 122: 532–541. 10.1037/a0031742 23421528

[36] Valera EM, Brown A, Biederman J, Faraone SV, Makris N, Monuteaux MC, et al (2010) Sex differences in the functional neuroanatomy of working memory in adults with ADHD. Am J Psychiatry 167: 86–94. 10.1176/appi.ajp.2009.09020249 19884224 PMC3777217

[37] Valera EM, Faraone SV, Biederman J, Poldrack RA, Seidman LJ (2005) Functional neuroanatomy of working memory in adults with attention-deficit/hyperactivity disorder. Biol Psychiatry 57: 439–447. 15737657 10.1016/j.biopsych.2004.11.034

[38] Quinlan DM, Brown TE (2003) Assessment of short-term verbal memory impairments in adolescents and adults with ADHD. J Atten Disord 6: 143–152. 12931072 10.1177/108705470300600401

[39] Toplak ME, Rucklidge JJ, Hetherington R, John SC, Tannock R (2003) Time perception deficits in attention-deficit/ hyperactivity disorder and comorbid reading difficulties in child and adolescent samples. J Child Psychol Psychiatry 44: 888–903. 12959497 10.1111/1469-7610.00173

[40] Faul F, Erdfelder E, Lang AG, Buchner A (2007) G*Power 3: a flexible statistical power analysis program for the social, behavioral, and biomedical sciences. Behav Res Methods 39: 175–191. 17695343 10.3758/bf03193146

[41] Retz-Junginger P, Retz W, Blocher D, Weijers HG, Trott GE, Wender PH, et al (2002) [Wender Utah rating scale. The short-version for the assessment of the attention-deficit hyperactivity disorder in adults]. Der Nervenarzt 73: 830–838. 12215873 10.1007/s00115-001-1215-x

[42] Rösler MRW, Retz-Junginger P, Thome J, Supprian T, Nissen T, Stieglitz R-D, et al (2004) Instrumente zur Diagnostik der Aufmerksamkeitsdefizit-/Hyperaktivitätsstörung (ADHS) im Erwachsenenalter: Selbstbeurteilungsskala (ADHS-SB) und Diagnose Checkliste (ADHS-DC). Nervenarzt 75: 888–895 15378249 10.1007/s00115-003-1622-2

[43] Laux PG, Schaffner P, Spielberger CD (1981) State-Trait-Angstinventar (STAI), Theoretische Grundlagen und Handanweisungen. Wenheim: Beltz Testgesellschaft.

[44] Beck AT, Steer RA, Brown GK (1996) Beck Depression Inventory-II San Antonio, TX: Harcourt Brace.

[45] Patton JH, Stanford MS, Barratt ES (1995) Factor structure of the Barratt impulsiveness scale. J Clin Psychol 51: 768–774. 8778124 10.1002/1097-4679(199511)51:6<768::aid-jclp2270510607>3.0.co;2-1

[46] Von Aster M. NAuHR (2006) Wechsler Intelligenztest für Erwachsene (WIE) Deutschsprachige Bearbeitung und Adaptation des WAIS-III von David Wechsler. Frankfurt: Harcourt Test Services.

[47] Lehrl S (1995) Manual zum MWT-B (Mehrfach-Wortschatz-Intelligenztest- Version B, 3. überarb. Aufl.). Balingen: Perimed.

[48] Härting C, Markowitsch H-J, Neufeld H, Calabrese P, Deisinger K, Kessler J (2000) Wechsler Memory Scale—Revised Edition, German Edition. Bern: Huber.

[49] Advokat C, Martino L, Hill BD, Gouvier W (2007) Continuous Performance Test (CPT) of college students with ADHD, psychiatric disorders, cognitive deficits, or no diagnosis. J Atten Disord 10: 253–256. 17242420 10.1177/1087054706292106

[50] Alderson RM, Kasper LJ, Hudec KL, Patros CH (2013) Attention-deficit/hyperactivity disorder (ADHD) and working memory in adults: a meta-analytic review. Neuropsychology 27: 287–302. 10.1037/a0032371 23688211

[51] Kasper LJ, Alderson RM, Hudec KL (2012) Moderators of working memory deficits in children with attention-deficit/hyperactivity disorder (ADHD): a meta-analytic review. Clin Psychol Rev 32: 605–617. 10.1016/j.cpr.2012.07.001 22917740

[52] Swanson JM (2003) Role of executive function in ADHD. J Clin Psychiatry 64 Suppl 14: 35–39. 14658934

[53] Barkley RA (1997) Behavioral inhibition, sustained attention, and executive functions: constructing a unifying theory of ADHD. Psychol Bull 121: 65–94. 9000892 10.1037/0033-2909.121.1.65

[54] Barkley RA (2010) Differential diagnosis of adults with ADHD: the role of executive function and self-regulation. J Clin Psychiatry 71: e17 10.4088/JCP.9066tx1c 20667287

[55] Barkley RA (2013) Distinguishing sluggish cognitive tempo from ADHD in children and adolescents: executive functioning, impairment, and comorbidity. J Clin Child Adolesc Psychol 42: 161–173. 10.1080/15374416.2012.734259 23094604

[56] Brown A, Biederman J, Valera E, Lomedico A, Aleardi M, Makris N, et al (2012) Working memory network alterations and associated symptoms in adults with ADHD and Bipolar Disorder. J Psychiatr Res 46: 476–483. 10.1016/j.jpsychires.2012.01.008 22272986 PMC3686289

[57] Fassbender C, Schweitzer JB, Cortes CR, Tagamets MA, Windsor TA, Reeves GM, et al (2011) Working memory in attention deficit/hyperactivity disorder is characterized by a lack of specialization of brain function. PLoS One 6: e27240 10.1371/journal.pone.0027240 22102882 PMC3213127

[58] Genro JP, Kieling C, Rohde LA, Hutz MH (2010) Attention-deficit/hyperactivity disorder and the dopaminergic hypotheses. Expert Rev Neurother 10: 587–601. 10.1586/ern.10.17 20367210

[59] Oades RD (2008) Dopamine-serotonin interactions in attention-deficit hyperactivity disorder (ADHD). Prog Brain Res 172: 543–565. 10.1016/S0079-6123(08)00926-6 18772050

[60] Ko CH, Yen JY, Yen CF, Chen CS, Lin WC, Wang PW, et al (2013) Brain activation deficit in increased-load working memory tasks among adults with ADHD using fMRI. Eur Arch Psychiatry Clin Neurosci 263: 561–573. 10.1007/s00406-013-0407-2 23645101

[61] Mette C, Zimmermann M, Grabemann M, Abdel-Hamid M, Uekermann J, Biskup CS, et al (2013) The impact of acute tryptophan depletion on attentional performance in adult patients with ADHD. Acta Psychiatr Scand 128: 124–132. 10.1111/acps.12090 23419004

[62] Schweitzer JB, Hanford RB, Medoff DR (2006) Working memory deficits in adults with ADHD: is there evidence for subtype differences? Behav Brain Funct 2: 43 17173676 10.1186/1744-9081-2-43 PMC1762010

[63] Ko CH, Hsieh TJ, Wang PW, Lin WC, Chen CS, Yen JY (2015) The Altered Brain Activation of Phonological Working Memory, Dual Tasking, and Distraction Among Participants With Adult ADHD and the Effect of the MAOA Polymorphism. J Atten Disord. 10.1177/1087054715572609 25777072

[64] Mullins C, Bellgrove MA, Gill M, Robertson IH (2005) Variability in time reproduction: difference in ADHD combined and inattentive subtypes. J Am Acad Child Adolesc Psychiatry 44: 169–176. 15689730 10.1097/00004583-200502000-00009

[65] Rattat AC, Droit-Volet S (2012) What is the best and easiest method of preventing counting in different temporal tasks? Behav Res Methods 44: 67–80. 10.3758/s13428-011-0135-3 21789731

